# Study on the Dynamics of Cortisol Secretions in Hypertensive Elderly Patients

**DOI:** 10.1155/2012/791412

**Published:** 2012-01-24

**Authors:** Doina Carstea, Diana-Maria Trasca, A. P. Carstea, E. T. Trasca

**Affiliations:** ^1^Department of Clinical Cardiology, Municipal Clinical Hospital “Phylantropia”, University of Medicine and Pharmacy, 200349 Craiova, Romania; ^2^Department of Internal Medicine, Municipal Clinical Hospital “Phylantropia”, University of Medicine and Pharmacy, 200349 Craiova, Romania; ^3^Department of Internal Medicine, The Research Centre of the Emergency Clinical Hospital, University of Medicine and Pharmacy, 200349 Craiova, Romania; ^4^Military Clinical Hospital Craiova, University of Medicine and Pharmacy, 200349 Craiova, Romania

## Abstract

Ageing is defined as a slow, irreversible process of cellular changes, that are due to a lack of balance between degradation and repair, a continuous interaction between physiological and pathological processes. Physiological aspects in elderly people are often confused with disease. Given these general considerations, we would make observations about the dynamics of cortisol secretion in healthy elderly subjects and patients with a diagnosed cardiovascular disease, more precisely hypertension. The study was conducted during 2003–2010, on a number of 135 patients older than 65 years of age, who were divided into two groups: one group counting 66 patients and consisting of healthy elderly controls (without systemic disease, renal, endocrine, or cardiovascular known issues) and group 2 who consists of 69 elderly patients who associate known hypertensive and other cardiovascular issues.

## 1. Introduction

Ageing is a worldwide recognized fact, implying social and economic aspects, wich lay in the way ageing is controled, because it could have an enormous impact regarding healthcare, hospitalization, and/or reconstructive surgery costs. Ageing is a degrading, progressive, inward-oriented, universal process, and the threshold between physiological and pathological is very thin [1]. Although the general approach towards elderly patients is to increase the quality of life (free prescriptions, occupational therapy, medical healthcare at home), there are a lot of real and recognized situations when the ageing phenomenon may lead to discrimination that can range from providing poor medical healthcare to restricted accessibility to some healthcare programs (e.g., screening for breast cancer in women over 65). However, we must be aware that the ageing process is in steady growth in all developed countries for two main reasons: on the one hand due to reduced mortality and on the other due to decreasing tendency of birth rate and fertility.

 Aging is defined as being a slow, irreversible changing process that occurs within single cells—the seat of some remarkable biochemical activity, whose development is genetically programmed in advance [2]. The notion of time in biology equals a measurement of motion in the form of rhythms and cycles, of which the circadian biorhythms of the endocrine functions are the most important. Variations in normal hormonal secretions differ from one hormone to another in the course of 24 hours, so ACTH and cortisol levels reach their peaks during early morning [3]. One of the most well-spread theories regarding aging is based on neuroendocrinology [4], and it well suits the global concept stating the lack of balance between degradation and repair, balance influenced by many internal and external factors [5].

 Circadian rhythm of cortisol secretion is stable, with little influence from light and sleep, and it is also highly reproductibile. It “matures” around the age of 4, remaining unchanged into elder years, with no difference in the two sexes. The rhythm of cortisol secretion is dependent on the circadian secretion of ACTH, its acrophase being around 4 AM. Feedback on the hypothalamic-pituitary axis, is exerted by cortisol on the autonomous circadian rhythm [6]. Hormones are secreted periodically, in a pulsatory manner. Hormonal secretion fluctuations may vary compared to time of day, month, or year. The rhythm is defined by a peak between the hours of 6–8 AM, the lowest value being recorded in the evening [7]. If more determinations of hormone levels are analysed, an ultradian rhythm is revealed, with a daily pulsatile secretion which reflects the episodic secretion of the zona fasciculata, in four phases over night: before bed, low cortisol secretion; during sleep, between 3–5 AM, higher secretion levels; from the last hours of sleep and the first hour after awakening, maximum discharge; during activity periods, intermittent discharge [8]. Clinical and paraclinical evaluation of the degree of impaired hormonal secretions in diseases—general and endocrine disorders—open new perspectives that will optimize chronomodulated therapeutic methods and means in relation to biological rhythms [9].

 Starting from these general considerations, we wanted to make some observations on the dynamics of cortisol secretion in healthy elderly people and those with known cardiovascular disease, especially those with hypertension.

## 2. Methods

The study was conducted in two locations: the Municipal Clinical Hospital “Philanthropy” and the Emergency Clinical County Hospital in Craiova during 2003–2010, with both a prospective and a retrospective component. Case studies presented are based on a number of 135 patients, over 65 years of age, who were divided into two groups: group one (SUBSET 1) consisting of 66 healthy elderly people (control group), without systemic, renal, endocrine, or cardiovascular known issues at the time of enrollment in the group and group 2 (SUBSET 2) consisting of 69 elderly patients who associate diagnosed hypertensive disease and other cardiovascular issues (ischemic heart disease, degenerative valvular heart disease, or arrhythmias, all of the patients being in compensated stages of heart failure—grade I or II NYHA, without any endocrine disorders). Of these, only 41 patients can be considered as “true” hypertensive patients, because the hypertension was the dominant issue compared to the others associated problems.

 The cases were investigated by means of clinical examination and cardiovascular evaluation carried out on standard protocol, supplemented by laboratory tests and further investigation (hematological, biochemical, enzymatic, hormonal, immunological, and imaging tests). Blood pressure measurement was made under the same circumstances in all subjects in the morning and evening, in supine and standing positions. Blood pressure was also measured in both arms due to the presence of atherosclerotic lesions, which in the elderly patients can lead to variations in the levels of the blood pressure values. Measurements were recorded for three consecutive days, and each individual patient's values was averaged to determine systolic and diastolic blood pressure. The WHO criteria for defining hypertension were commonly used. We considered as significant for the inclusion in the category of hypertensive patients, values higher than 140 mm Hg (regarding systolic pressure), and also values higher than 80 mm Hg (regarding the diastolic values). In order to be able to correctly classify hypertension, in addition to blood pressure measurement values, we aimed to assess whether or not target organ damage could be recorded, optic blood vessels were analysed, ECG and echocardiography (for left ventricular hypertrophy) were performed, as well as urinary protein dosage.

 Patients in both groups were put through further hematological and biochemical sorting investigations, along with serum cortisol determination. The dosage system “Elecys 1010” found in the laboratory of the Emergency Clinical County Hospital of Craiova was used to measure cortisol levels. The sorting and assessment of trial groups were completed by specific-cardiovascular oriented investigations (echocardiography and electrocardiogram) and the complex imaging explorations (radiology, ultrasound, and MRI).

 The results were processed by statistical methods (Student's *t*-test, Bartlett's test, Kruskal-Wallis' test, Chi-squared test, the arithmetic mean, standard deviation, ANOVA test, and simple linear regression), using the “Data Analysis” subpackage module Microsoft Excel program and also the “EP12000” program, specialized in the execution of graphs, tables, and statistical tests.

 In this study, we will use data from the measurements of cortisol levels, with normal values admitted to this study for cortisol serum levels in the morning (measured between 7–10 AM) ranging between 171 and 536 nmol/L (cortisol 1), while cortisol values in the evening (measured between the hours of 6–8 PM) range between 64 and 327 nmol/L (cortisol 2).

## 3. Results

In this study, we analyzed two groups of patients in order to observe the dynamics of hormonal secretion in elderly patients: a control group (subset 1) consisting of 66 healthy elderly people and a study group (subset 2) consisting of 69 elderly patients diagnosed with hypertensive disease associated with various cardiovascular diseases. The percentage analysis of cases grouped by age showed that the largest share of older people in both groups belonged to age group of 75–84 years. The average values of biochemical and hematological parameters were within normal limits of age in both groups, except for some values of glucose or lipid fractions.

*Subset 1* or the control group was represented by healthy elderly, who had been submitted for routine clinical and laboratory tests. Following the recollection of past medical history, anamnesis, clinical examination, and laboratory tests, 66 healthy elderly people have been selected for the study. The subset was composed of people over 65 years of age (limits between 65 and 92 years), mean age being 78.31 in men and 78.53 in women. Three age groups have been made: first age group ranging between 65 and 74, second one ranging from 75 to 84, and the last group for patients older than 84. The group's structure included more men (40 male cases) than women (26 cases), sex ratio equaling 1.53 in the studied group.

Cortisol 1 values were measured in the morning, between 7 and 8 AM. Mean cortisol measured at this time among the cases in SUBSET 1 was 336.92 nmol/L (limits at 172–537 nmol/L, CI95% 306.47–367.38 nmol/L). A statistically significant difference between mean values of cortisol 1 was not recorded in subjects from subset 1 regarding age differences (*P* = 0.168), but mean cortisol 1 was higher in women (mean 396.38 nmol/L; limits 266–536 nmol/L, CI95% 360.16–432.61 nmol/L) than in men (mean 298.27 nmol/L, limits 172–537 nmol/L, CI95% 257.14–339.4 nmol/L) with about 100 nmol/L. *t*-test to compare mean values identified a high statistical significance for the difference between the two areas (*P* = 0.0012).

Cortisol 2 values were measured in the evening, between 6 and 8 PM. An average value of 205.58 nmol/L (limits 67–327 nmol/L; CI95% 186.27–224.88 nmol/L) was identified in all the subjects in group 1. Measurements in the evening recorded no significant variation regarding age groups in the healthy eldery (*P* = 0.09), but mean cortisol 2 was higher in women (mean 238.81 nmol/L, limits 144–317 nmol/L, CI95% 217.27–260.34 nmol/L) than in men (mean 183.97 nmol/L, limits 67–327 nmol/L, CI95% 156.76–211.19 nmol/L), with high statistical significance for the difference between the two areas (*P* = 0.0047).

*Subset 2* consisted of elderly patients with hypertension and cardiovascular disease, but not endocrine issues. These patients were admitted for previously diagnosed cardiovascular issues or newly diagnosed disease. Following the anamnesis, physical examination, and laboratory findings, a total of 69 patients were selected for the study. Similar to subset 1, subset 2 was composed of elderly over 65 years of age (ages ranging from 65 to 90 years); the patients were also divided into three age groups the same as group 1. Men were at a slight advantage (40 cases—58%) over women (29 cases—42%), sex ratio equaling 1.38 in the studied group. Women taken into study from this group had ages between 65 and 89 years of age (mean age 77.69); the men had ages ranging from 65 to 90 years old (mean age 78.22).

Mean cortisol 1 measured at 8 am, in subjects included in group 2, was 392.93 nmol/L (limits 172–536 nmol/L, CI95% 363.66–422.19 nmol/L), while average cortisol 2 measured at 8 PM, among the cases in group 2, was 228.55 nmol/L (limits 73–326 nmol/L, CI95% 210.58–246.52 nmol/L). There was no statistically significant link between the age of the subjects of subset 2 and cortisol values 1 or 2.

Knowing that, in the elderly multiple pathology is very frequent; it was quite difficult to select patients with one cardiovascular disease. Thus, we have selected for the study the most frequently encountered heart diseases in clinical practice: high blood pressure (59%), ischemic heart disease (58%), heart failure (29%), and fibrillation (28%). The associations between the above-mentioned issues are as follows: first pairing was represented by hypertension—ischemic heart disease (17 cases, 24.6%), followed by the association of hypertension-heart failure (11 cases, 15.9%), hypertension and atrial fibrillation (5 cases, 7.2%), and finally hypertension, ischemic heart disease, heart failure, and atrial fibrillation (3 cases, 4.3%).

For these reasons we have formed a group of patients called *subset 2′*, with cardiovascular issues, but with clinical dominating hypertension symptoms, consisting of 41 subjects.

### 3.1. A Comparative Analysis of Cortisol 1 in the Two Groups

Mean cortisol 1, measured within the hypertension dominant group (subset 2′), was 483.73 nmol/L (limits 321–536 nmol/L, CI95% 471.46–496.01 nmol/L).

 Comparing the mean morning cortisol in the control group (336.92 nmol/L) to that obtained in patients from subset 2′, we noticed that the average value is higher for the latter (483.73 nmol/L); the difference between the two values being highly significant in statistical terms (*P* < 0.001) (Table 1).

**Figure 1:**
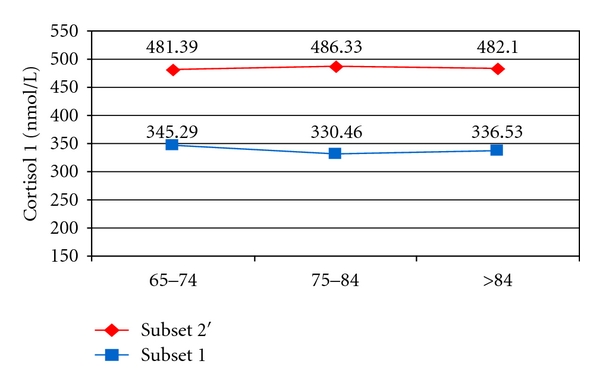
Mean cortisol 1 in subset 1 and 2′ by age groups.

 By groups of age, mean cortisol 1 values were over 130 nmol/L higher in hypertensive subjects compared with the subjects in group 1, regardless of age pairings. A mean cortisol 1 was averaging at about 480 nmol/L in group 2′, while the subjects in group 1 did not record a value higher than 346 nmol/L (Figure 1). 

 Regardless of the grading of the hypertensive disease, patients with hypertension in group 2′ had an average value of cortisol 1 higher than the one recorded in the control group. 

**Figure 2:**
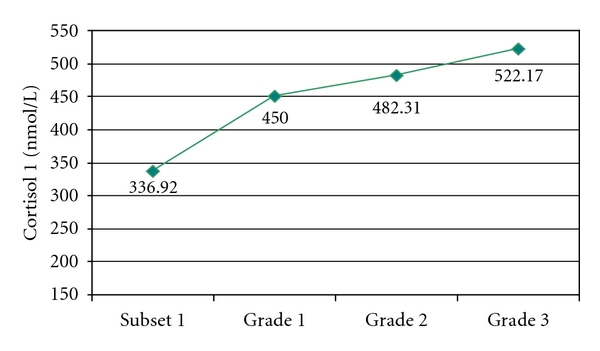
Mean cortisol 1 value in subset 1 and in hypertensive subjects.

 Also, as the severity of the hypertensive disease was greater, mean cortisol 1 was higher, the difference being highly significant (*P* < 0.001). Thus, the subjects in subset 1 had a mean cortisol 1 of 336.92 nmol/L, while patients' cortisol 1 value (from subset 2′) increased proportional to the grading of the hypertensive disease (grade 1—450 nmol/L, grade 2—482.31 nmol/L, and grade—522.17 nmol/L) (Figure 2). 

### 3.2. A Comparative Analysis of Cortisol 2 in the Two Groups

Cortisol 2 measured at 8 PM averaged at 205.58 nmol/L in subset 1 (limits 67–327 nmol/L; CI95% 186.27–224.88 nmol/L). In subset 2, the average value of cortisol measured at 8 PM was higher—228.55 nmol/L (limits 73–326 nmol/L; CI95% 210.58–246.52 nmol/L). The difference between the 2 obtained values was not significant in statistical terms. 

 Comparing the average value of cortisol 2 in subset 1 (205.58 nmol/L) with the one obtained from the subjects of subset 2′, we can clearly observe that the average value in hypertensive patients (281.42 nmol/L) is higher than the average value of cortisol 2 in the control group, this being highly significant (*P* < 0.001) (Table 2). 

**Figure 3:**
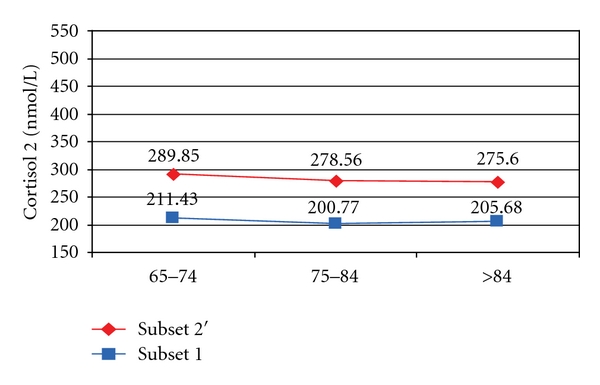
Mean cortisol 2 in patients from subsets 1 and 2′ by age groups.

 If we analyse by age group, mean values of cortisol 2 (measured in the evening) were about 70 nmol/L higher in group 2′ compared to the subjects of the control group in all age groups (Figure 3). 

**Figure 4:**
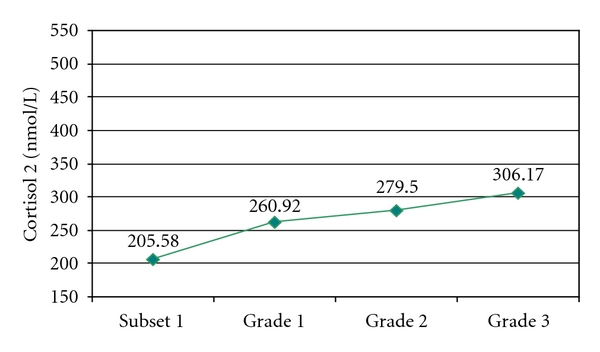
Mean cortisol 2 in subset 1 and 2′ by grade of hypertension.

 Regardless of the grade of the hypertensive disease, mean cortisol 2 values were higher than in group 1, and the more severe the hypertension was, the higher the mean value of cortisol 2 was. The difference recorded to the control group is highly significant in statistical terms (*P* < 0.001) (Figure 4). 

### 3.3. A Comparative Analysis of Cortisol 1 and 2 Values in the 2 Study Groups

**Figure 5:**
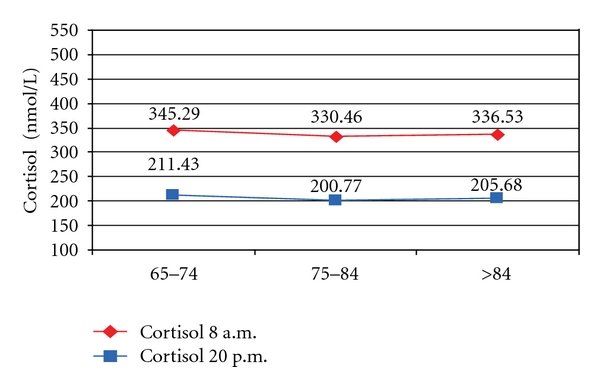
Dynamics of cortisol values in group 1.

For group 1 the mean cortisol 1 value was 336.92 nmol/L, and for cortisol 2 a mean value of 205.58 nmol/L was identified. We can clearly say that in control group subjects, for all age groups, mean cortisol 2 values were about 130 nmol/L lower than those recorded during the mornings (Figure 5). 

**Figure 6:**
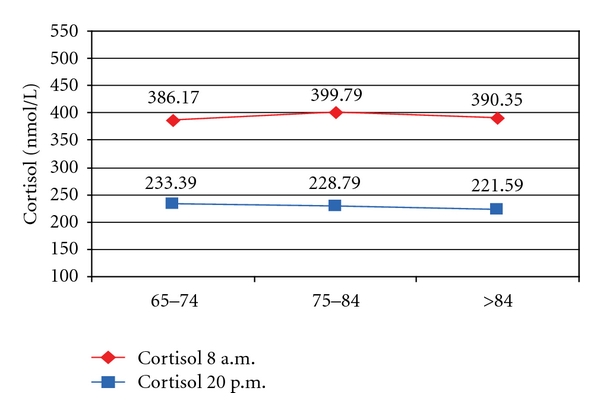
Dynamics of cortisol values in group 2.

 Mean morning cortisol measured in subjects enrolled in group 2 was 392.93 nmol/L, and mean cortisol value measured at 8 PM in the same group was 228.55 nmol/L. Just like in the case of the control group, we can clearly state that the patients with cardiovascular issues, in all age groups, have mean values of cortisol 2 lower than the morning value with over 150 nmol/L (Figure 6). 

 Patients in group 2 had a more pronounced decrease in cortisol 2 values than in cortisol 1 (over 150 nmol/L), compared with the decrease recorded in the control group (about 130 nmol/L). 

 The patients in group 2 recorded a mean cortisol 1 value higher (392.93 nmol/L) than the one of the control group (336.92 nmol/L), the difference between the two values being statistically significant (*P* = 0.009). 

 Although average values of cortisol 2 were higher in patients with cardiovascular disease (228.55 nmol/L) compared with the values obtained in the control group (205.58 nmol/L), the difference was not significant in statistical terms. 

**Figure 7:**
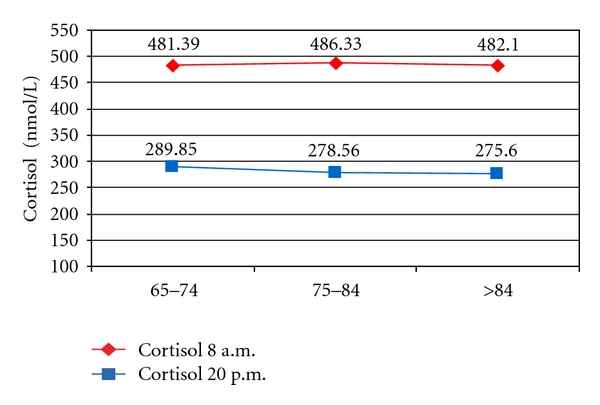
Dynamics of cortisol values in hypertensive patients.

 For group 2′ (cardiovascular afflicted patients, with dominating hypertension symptoms), mean morning cortisol was 483.73 nmol/L and the evening cortisol in the same patients was 281.42 nmol/L. In hypertensive patients, mean cortisol 1 values, by age groups, were similar, ranging between 481 and 486 nmol/L, to cortisol 2 values which ranged between 275 and 289 nmol/L (Figure 7). 

**Figure 8:**
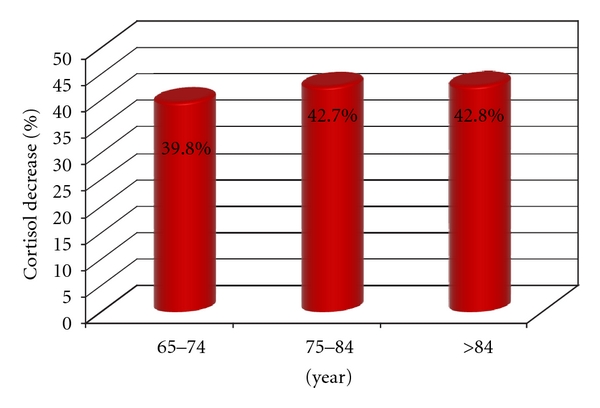
Percentage decrease in cortisol levels, by age groups, in group 2′.

 Decrease in cortisol 2 values in patients with hypertension was around 200 nmol/L to morning cortisol in all age groups. Comparing, the biggest difference between values of cortisol 1 and 2, in patients in group 2′, was recorded in the age groups of 75 to 84 and over 84 years (42.7% and 42.8%) (Figure 8). 

## 4. Discussion

The present study confirms some existing data in the literature, namely that, in the elderly, normal levels of cortisol are recorded. Because of purely economic reasons, multiple measurements of cortisol levels could not be performed in the same patient. 

 In some studies, normal values of cortisol in elder patients have been recorded, but with a decrease in the amplitude of its diurnal variations [10]; slightly elevated values have also been recorded [11], especially in men with cardiovascular diseases [12], resistance to cortisol suppression also being noticed in other studies [13]. 

 In this study, the dynamics of cortisol was within normal reference limits, both in healthy elderly and in elderly hypertensive patients, with occasional narrowing or widening of the range of obtained results. 

 We noted that healthy women over 65 years in group 1 had higher mean cortisol levels than average elderly men from the same set. 

 Elderly hypertensive patients had mean cortisol values greater than those obtained in healthy people aged over 65 years. It is highly important that the mean values of cortisol, regardless of when they were determined (morning or evening), varied along with the severity of blood hypertension: the higher the degree of hypertension was, the higher the mean cortisol value was. 

 Patients with cardiovascular disease, especially hypertensive patients, showed a more pronounced decrease of evening determined cortisol values, compared with healthy elderly.

## 5. Conclusion

1. Mean morning and evening cortisol measured in both groups were within normal limits, approaching the upper limit of reference.
2. Mean morning and evening cortisol determined were higher in healthy women compared with men from the same group.
3. The mean values of morning and evening cortisol recorded in hypertensive patients were higher than the values obtained from healthy patients.
4. The severity of blood hypertension correlated with cortisol values: the higher the grading of hypertension was, the higher mean cortisol determined during morning or evening was.
5. We take into account the need to introduce the screening of hormonal levels (specifically cortisol values) in elderly patients, because the modified values of certain hormones may become predictive factors for the evolution and prognosis of cardiovascular diseases.

## Figures and Tables

**Table 1:** Mean cortisol 1 in patients from subset 1 and 2′.

Cortisol 1	*N*	Average	CI95%	SD	Minimum	Maximum	*P*
Subset 1	66	336.92	306.47–367.38	123.89	172	537	<0.001
Subset 2′	41	483.73	471.46–496.01	38.89	321	536	

**Table 2:** Mean cortisol 2 in patients from subset 1 and 2′.

Cortisol 2	*N*	Average	CI95%	SD	Minimum	Maximum	*P*
Subset 1	66	205.58	186.27–224.88	78.54	67	327	<0.001
Subset 2′	41	281.42	270.76–292.07	33.74	194	326	

## References

[1] Merck HB, Berkow R (2000). *The Merck Manual of Geriatrics*.

[2] Ladisls R (2000). Cellular and molecular mechanisms of aging and age related diseases. *Pathology and Oncology Research*.

[3] Buckley TM, Schatzberg AF (2005). Aging and the role of the HPA axis and rhythm in sleep and memory-consolidation. *American Journal of Geriatric Psychiatry*.

[4] Baranowska B, Wolinska-Witort E, Bik W, Baranowska-Bik A, Martynska L, Chmielowska M (2007). Evaluation of neuroendocrine status in longevity. *Neurobiology of Aging*.

[5] Smith RG, Betancourt L, Sun Y (2005). Molecular endocrinology and physiology of the aging central nervous system. *Endocrine Reviews*.

[6] Becker KL (2002). Endocrinology and aging. *Principles and Practice of Endocrinology & Metabolism*.

[7] Kripke DF, Youngstedt SD, Elliott JA (2005). Circadian phase in adults of contrasting ages. *Chronobiology International*.

[8] van Cauter E, Leproult R, Kupfer DJ (1996). Effects of gender and age on the levels and circadian rhythmicity of plasma cortisol. *Journal of Clinical Endocrinology and Metabolism*.

[9] Vgontzas AN, Zoumakis M, Bixler EO (2003). Impaired nighttime sleep in healthy old versus young adults is associated with elevated plasma interleukin-6 and cortisol levels: physiologic and therapeutic implications. *Journal of Clinical Endocrinology and Metabolism*.

[10] Valenti G (2002). Adrenopause: an imbalance between dehydroepiandrosterone (DHEA) and cortisol secretion. *Journal of Endocrinological Investigation*.

[11] Chehab O, Ouertani M, Chaieb K, Haouala F, Mahdouani K (2007). Hormonal status of cortisol and dehydroepiandrosterone sulfate in an elderly Tunisian population. *Comptes Rendus Biologies*.

[12] Zhao ZY, Lu FH, Xie Y, Fu YR, Bogdan A, Touitou Y (2003). Cortisol secretion in the elderly. Influence of age, sex and cardiovascular disease in a Chinese population. *Steroids*.

[13] Carvalhaes-Neto N, Huayllas MK, Ramos LR, Cendoroglo MS, Kater CE (2003). Cortisol, DHEAS and aging: resistance to cortisol suppression in frail institutionalized elderly. *Journal of Endocrinological Investigation*.

